# Markerless inside-out tool tracking for endoscopic spine surgery: a benchmarking study and clinical dataset

**DOI:** 10.1007/s11548-026-03625-z

**Published:** 2026-04-27

**Authors:** Peter Zhang, Youyang Shen, Frédéric Giraud, Nicola A. Cavalcanti, Marc Pollefeys, Christoph J. Laux, Philipp Fürnstahl, Hooman Esfandiari

**Affiliations:** 1 Computer Vision and Geometry Group, ETH Zurich, Zurich, Switzerland; 2 Research in Orthopedic Computer Science, University Hospital Balgrist, University of Zurich, Zurich, Switzerland; 3 X23D AG, Zurich, Switzerland; 4 Spatial AI Lab, Microsoft, Zurich, Switzerland; 5 Balgrist University Hospital, University of Zurich, Zurich, Switzerland

**Keywords:** Tool tracking, SLAM, Surgical navigation, Camera pose estimation

## Abstract

**Purpose:**

Markerless inside-out surgical instrument tracking using a tool-mounted camera offers a promising solution to the limited clinical adoption of the existing navigation systems, which primarily rely on outside-in optical tracking and are constrained by line-of-sight issues. However, its performance in the surgical environment, with its unique challenges, remains largely unexplored. This work benchmarks state-of-the-art inside-out methods, namely, visual Simultaneous Localization and Mapping (vSLAM) methods. To this end, we collected a first-of-its-kind dataset in spine endoscopy, providing ground-truth tool poses.

**Methods:**

We recorded endoscopic spine surgeries performed on a high-fidelity training model in a real operating room environment, containing synchronized stereo images from tool-mounted cameras, sub-millimetric ground truth pose data from a commercial optical tracking system, and the endoscopic feed. Using this dataset, the instrument tracking accuracy of a selected number of vSLAM algorithms was compared.

**Results:**

The best performing approach achieved a root mean squared absolute trajectory error of 2.0 mm and 1.47 degrees, reaching accuracies of around 1 mm and 1 degree on selected sequences. However, it shows degraded performance in the presence of challenges such as occlusions and scene-object dynamics.

**Conclusions:**

Markerless inside-out tracking using vSLAM demonstrated high accuracy, indicating potential feasibility for navigated endoscopic spine applications. Our evaluation revealed that current algorithms remain insufficiently robust for routine clinical use. The presented study and dataset establish a foundation for future research toward reliable, real-time inside-out navigation in minimally invasive surgery.

**Supplementary Information:**

The online version contains supplementary material available at https://doi.org/10.1007/s11548-026-03625-z.

## Introduction

Real-time surgical tool tracking is paramount in computer and robotic-assisted surgeries, enabling intraoperative navigation through accurate surgical guidance. Procedures such as newly emerging minimally invasive endoscopic spine surgery [1, 2] could benefit substantially from reliable tool tracking. Compared to conventional fluoroscopy-guided endoscopic targeting, surgical navigation systems have been shown to improve the learning curve associated with spinal endoscopy [3–5], which could boost the clinical adoption rate. However, established navigation methods are based on electromagnetic or marker-based optical tracking technologies [6], both of which have workflow limitations. Electromagnetic tracking is highly sensitive to metallic objects, and optical tracking (i.e., outside-in tracking) comes with restrictive line-of-sight constraints [7].

To address these limitations, marker-less surgical tool tracking has recently gained traction as an emerging research direction in the broader computer-assisted surgery community [8–10]. Yet, most developments in this area rely on outside-in setups via static peripheral camera(s) that detect and track the surgical tools. These setups suffer from the same drawbacks as the marker-based techniques, hindering clinical adoption. In contrast, inside-out tracking presents a viable alternative for procedures such as endoscopic spine surgery to address the said challenges. By using surgical tool-mounted camera(s), the 3D pose of the surgical tool could be tracked throughout the surgery, potentially offering a cost-effective tracking method with less severe line-of-sight constraints.

Building upon this motivation, our study aims to advance the field by introducing, to the best of our knowledge, the first explorations of inside-out tracking for spinal endoscopy under realistic surgical conditions. We designed an inside-out tracking prototype by mounting lightweight stereo cameras onto surgical instruments and used them to capture a dedicated intraoperative dataset with realistic endoscopic workflows. It is publicly available on our project page https://peetzha.github.io/ess-io/. The main contributions of this work are therefore:

1. Presenting the first structured evaluation of state-of-the-art inside-out tracking methods in the surgical domain, and assessing their performance in spinal endoscopy.
2. Collecting and curating a dataset for evaluating inside-out tracking algorithms under realistic surgical conditions (e.g., field of view, operating room lighting, surgical motion, etc.).
3. Releasing this dataset as a public benchmarking resource for inside-out surgical tool tracking in endoscopic spine surgery, thus enabling future developments.

**Fig. 1:**
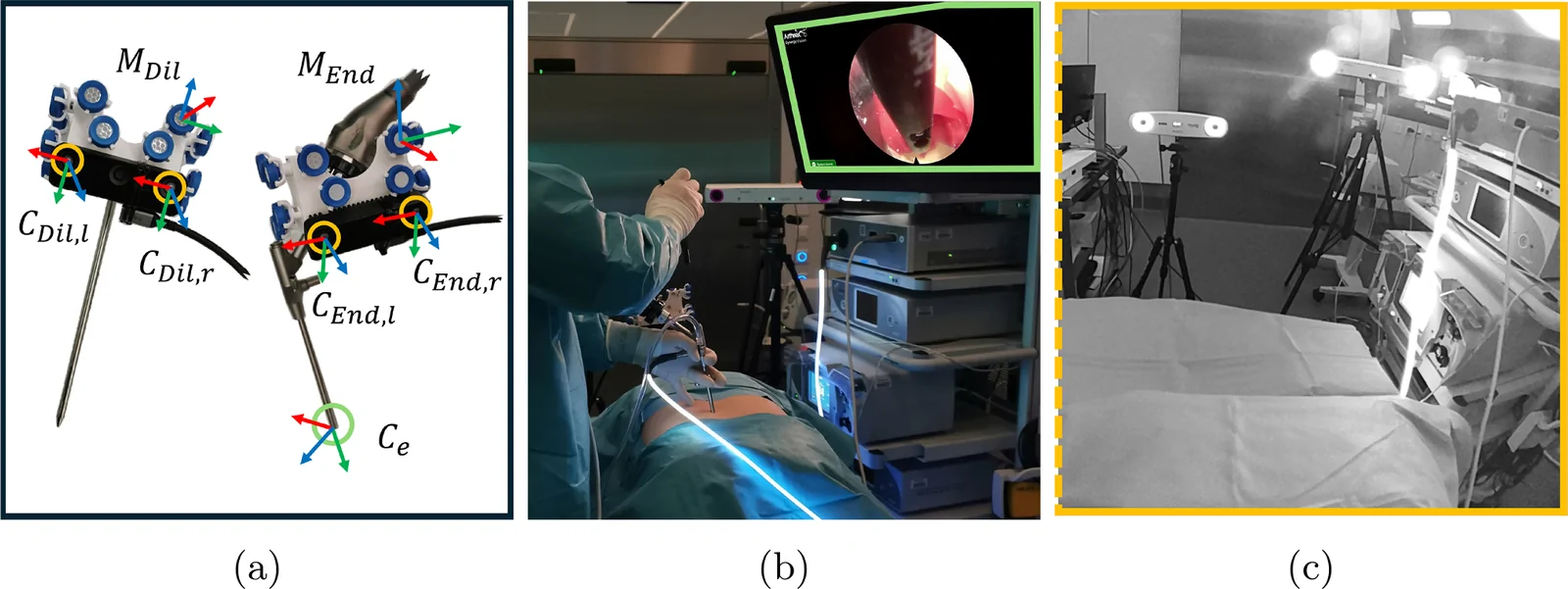
Inside-out tracking setup. **a** Dilator and endoscope with the mounted stereo cameras and blue retroflective markers. **b** Example endoscopic intervention phase of the recordings. **c** The cropped field of view of the endoscope-mounted stereo camera

## Related work

In this section, we present related works on marker-less surgical tool tracking, focusing mainly on inside-out tracking, before providing a short overview of the general state-of-the-art in the broader computer vision field.

Marker-less tool tracking aims to reduce the setup complexity of navigation systems. Outside-in approaches typically try to use a static RGB camera observing the surgical procedure to estimate tool poses [9–11]. While this reduces the preparation time, the line-of-sight constraint remains. The inside-out approach leverages camera views from a tracked object itself to estimate its pose within the environment, which has been applied in the medical context for virtual or augmented reality applications [12, 13]. Endoscopic and laparoscopic video-based tracking can be considered conceptually similar, representing an active area of ongoing research [14–16]. Such methods leverage the observed anatomy in the image feed to estimate the camera pose, a highly demanding task due to tissue deformations, challenging lighting conditions, and strong occlusions. These factors are especially pronounced in endoscopic spine surgery. The anatomy is observed through a narrow field of view, in which the surgeon continuously deforms and resects surrounding tissue, severely complicating consistent long-term tracking.

Thus, at this early stage, focusing on data collection and method development, we propose to mount an additional camera facing the operating room directly onto the surgical tool. A limited number of prior art have explored this concept. In [8], the authors attached a stereo camera on an ultrasound probe using the estimated poses for 3D ultrasound compounding of the prostate. They reported a root mean squared translation error of 2.65±0.74 mm when using ORB-SLAM2 [17]. A related study [18] achieved 0.85±0.50 mm 2D error when mounting a large baseline stereo camera onto a hand-held Optical Coherence Tomography (OCT) probe, but did not compare against a reliable ground truth. However, neither study provides details on the performed trajectories, nor were they performed under realistic surgical conditions. Both key factors that influence the expected tracking accuracy.

Therefore, a structured evaluation of state-of-the-art inside-out surgical tool tracking using established Simultaneous Localization and Mapping (SLAM) metrics in realistic surgical settings remains an open area of investigation and constitutes one of the primary motivations for the present study.

SLAM infers its own reference frame by extracting and tracking visual features in the environment, thus tackling the marker-less inside-out tracking problem. While some approaches can integrate a plurality of sensor modalities, vision-only configurations represent one of the least intrusive options for clinical workflows, offering simple initialization, compact hardware design, and low cost.

These visual SLAM (vSLAM) methods can be grouped into indirect, direct, and learning-based approaches. Indirect methods (e.g., ORB-SLAM2/3 [17, 19]) track sparse features, optimizing the geometric consistency, and are efficient but are sensitive to feature-poor conditions. Direct methods such as DSO [20] estimate pose by optimizing photometric error across selected image regions without explicit feature extraction. Recently, learning-based SLAM methods (DROID-SLAM [21], DPVO [22], MASt3R-SLAM [23]) have demonstrated superior robustness and accuracy by leveraging models predicting optical flow, depth, or 3D point clouds directly from frames, based on large datasets used for training.

In this study and for the particular use case of inside-out tracking in spine surgery, we aim to provide a structured comparative analysis of representative state-of-the-art in each of the afore-mentioned categories, namely: indirect feature-based SLAM (ORB-SLAM3), direct photometric SLAM (DSO), learning-based SLAM (DROID-SLAM, DVPO, and MASt3R-SLAM).

## Methodology

In this section, we describe the experimental setup and methodological framework of this study, covering sensor configuration and calibration, dataset acquisition and characteristics, and the benchmarking protocol used for evaluation.

### Sensor setup and calibration

An Oak-D Pro W (Luxonis, USA) stereo camera ($C_{i}$ for i∈(left,right)), chosen due to its form factor, ergonomics and potential modularity, was rigidly mounted on both the dilator (*Dil*) and the endoscope (*End*), as shown in Fig. 1. The cameras were mounted in a downward 30$^\circ$ orientation, identified as optimal in preliminary tests. The units were used as hardware-synchronized wide-angle global-shutter stereo cameras, with a 75 mm stereo baseline, recording at a resolution of 1280x800 px and a frame rate of 20 Hz. The endoscopic feed (Arthrex Synergy Vision, USA; 30$^\circ$ scope) was recorded at 1920x1080 px and 60 Hz. The ground truth tool and anatomy poses were obtained using a fusionTrack™500 (Atracsys, Switzerland) optical tracking system, tracking retroflective markers mounted on the instruments and surgical model.

**Fig. 2:**
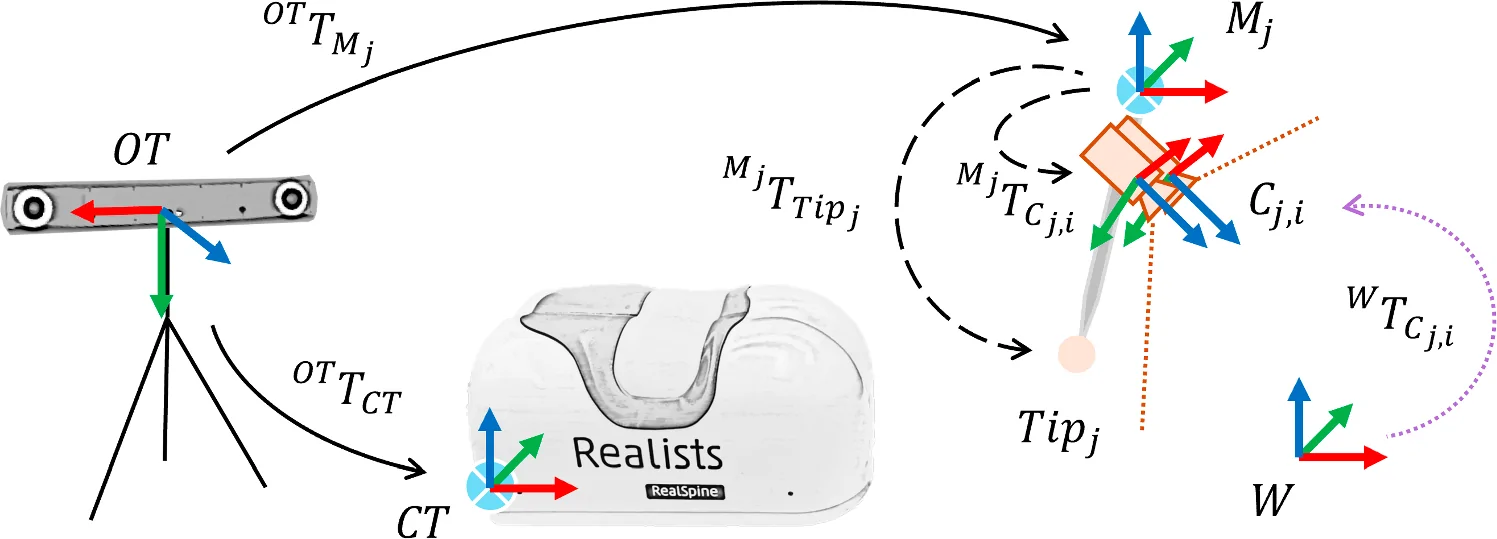
Illustration of the sensor system used to capture the dataset. Arrows indicate measured transformations, and dashed arrows calibrated or estimated ones

All cameras $C_{i,j}$ for j∈(Dil,End) were intrinsically and extrinsically calibrated using a printed ChArUco target and the methods provided by the manufacturer,^1^ resulting in mean reprojection and epipolar errors of 0.15 px and 0.19 px, respectively.

To provide an opportunity for future work in navigated spine surgery (e.g., real-time 3D navigation), we acquired a pre-operative CT scan of the model. The tracking system *OT* provides the transformations ${}^{OT}T_{M_j}$ and ${}^{OT}T_{CT}$ with sub-millimetric accuracy, enabling the tracking of a variety of transformations. A schematic overview of the provided transformations is shown in Fig. 2. By estimating the rigid transformation ${}^{M_j}T_{Tip_j}$ using pivot calibration, the typical transformation of interest for surgical navigation between the acquired pre-operative CT and each tool tip is expressed as:

1 $$\begin{aligned} {}^{CT}T_{Tip_j}(t)=[{}^{OT}T_{CT}(t)]^{-1} \cdot {}^{OT}T_{M_j}(t) \cdot {}^{M_j}T_{Tip_j} \end{aligned}$$

Using the rigid transformation ${}^{M_j}T_{C_{i,j}}$, we can chain the transformations to obtain the continuous ground truth trajectory required to compare SLAM methods:

2 $$\begin{aligned} {}^{OT}\bar{T}_{C_{i,j}}(t)={}^{OT}T_{M_j}(t) \cdot {}^{M_j}T_{C_{i,j}} \end{aligned}$$

We calibrated ${}^{M_j}T_{C_{i,j}}$ using the Perspective-n-Point (PnP) algorithm on a set of poses where a tracked planar checkerboard pattern (same as in [24]) is visible, with a mean reprojection error of 1.6 pixels. The details regarding the similar transformation chain for the endoscopic camera are given in the Online Resource 1.

All sensor systems were synchronized to a host computer clock. The Oak cameras synchronized their timestamps with sub-millisecond accuracy, while the optical tracking system was hardware synchronized using Precision Time Protocol (PTP), thus achieving microsecond-level alignment.

### Dataset

Our dataset comprised recorded sequences of four interlaminar discectomies performed by a board-certified spine surgeon on a state-of-the-art surgical training anatomical model (RealSpine Advanced MISS, Realists, Germany) with a high-fidelity representation of soft tissue structures. As in real surgery, continuous saline irrigation was used, and all procedures were performed in a real operating room, closely imitating the clinical setup, with the surgeon defining equipment placement and lighting conditions. The interventions targeted the levels L4-L5, with two procedures recorded from the left side and the other two from the right side of the patient model. The resulting dataset consisted of calibrated, co-registered, and synchronized recordings from the tool-mounted inside-out camera, the endoscopic camera, the ground-truth optical tracking system, and the corresponding registered preoperative CT scan.

To target the pathology, the surgeon used a rigid dilator, navigated via the integrated Realists RealImaging navigation system. Therefore, each recording followed a typical endoscopic surgical workflow, consisting of two distinct phases:

1. *Endoscopic targeting phase*, in which the dilator was employed (j=Dil)
2. *Endoscopic intervention phase*, in which the endoscope was used (j=End)

### Benchmarking

To evaluate monocular vSLAM performance for our target application, we used the right camera recordings to run DSO [20], ORB-SLAM3 [19], DROID-SLAM [21], DPVO [22], and MASt3R-SLAM [23] in monocular mode. Additionally, stereo variants of ORB-SLAM3 and DROID-SLAM were tested. These methods observe an unregistered environment *W*, providing continuous pose estimates ${}^{W}\tilde{T}_{C_{i,j}}(t)$, which we compare against the ground truth ${}^{OT}\bar{T}_{C_{i,j}}(t)$. We used Umeyama’s method [25], to align the frames *W* and *OT* such that:

3 $$\begin{aligned} {}^{OT}\tilde{T}_{C_{i,j}}(t)=s \cdot {}^{OT}\tilde{T}_{W} \cdot {}^{W}\tilde{T}_{C_{i,j}}(t), \end{aligned}$$

where *s* is a scaling factor used in the alignment, as pure monocular methods are unable to recover scale. Performance was evaluated using the root mean squared Absolute Trajectory Error (rms ATE), computed for both translation and rotation by comparing the aligned SLAM estimates ${}^{W}\tilde{T}_{C_{i,j}}(t)$ against the ground-truth trajectories ${}^{OT}\bar{T}_{C_{i,j}}(t)$. For each method, we report the median rms ATE over three independent runs using default parameters. ORB-SLAM3 was executed on an Intel Core Ultra 7 155 H, while all other methods were evaluated on a system equipped with an Intel Core i7-14700K and an NVIDIA RTX 4090. All methods exceeded 20 Hz, except MASt3R-SLAM, achieving approximately 17 Hz.

## Results

### Dataset

**Table 1:** Dataset overview. L and R denote the procedure performed on the model’s left and right side, while Dil and End are shorthand notations for the used tools

	Duration [s]	Motion blur	Dynamics	Occlusions (Lcam, Rcam)
Left1Dil	18.4	mild	background	none, none
Left1End	1139.8	negligible	background	partial, partial
Right1Dil	14.4	negligible	none	none, none
Right1End	931.7	negligible	foreground	severe, partial
Left2Dil	21.5	moderate	background	partial, partial
Left2End	1577.6	mild	negligible	frequent, partial
Right2Dil	26.2	mild	background	none, none
Right2End	1324.1	negligible	foreground	severe, partial

**Fig. 3:**
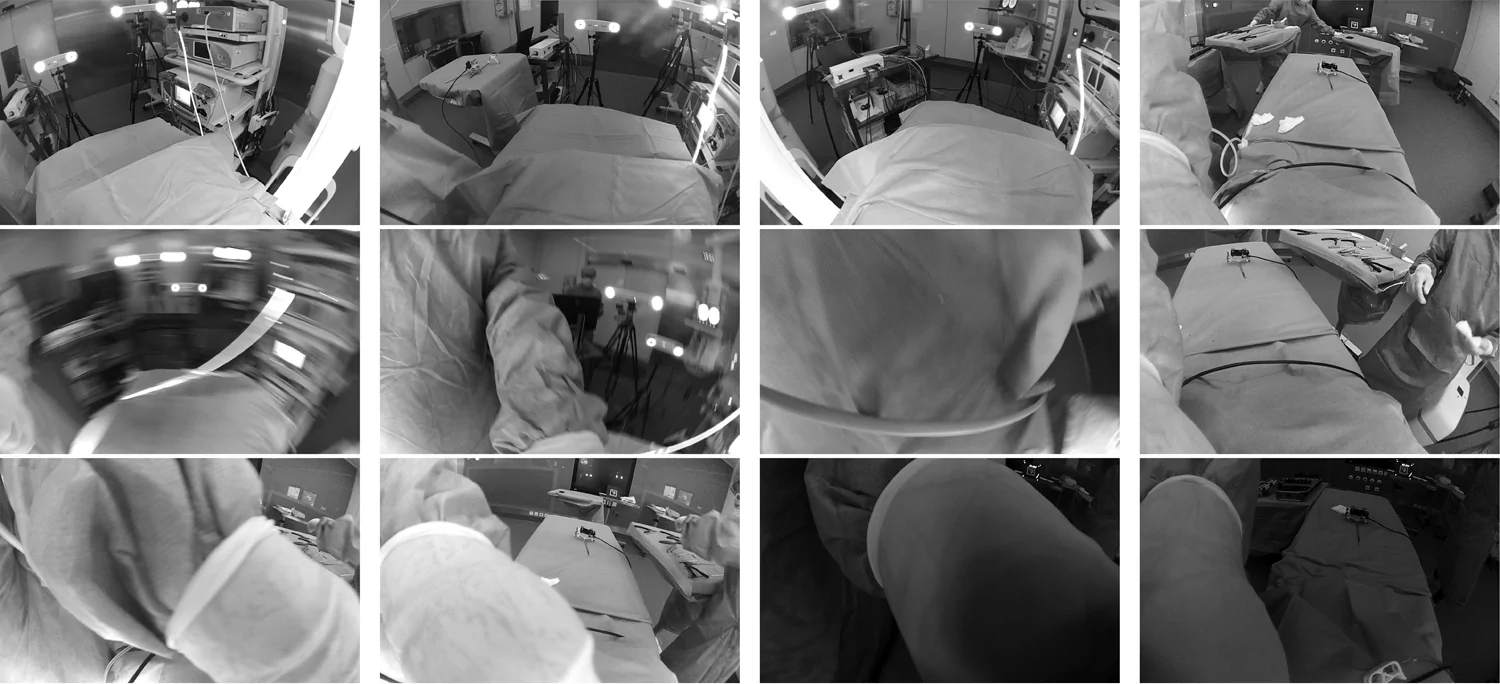
Top row: Sample frames from Left1Dil, Left1End, Right1Dil, Right1End. Middle row: Challenging example frames, depicting motion blur, partial occlusions, featureless regions, or scene dynamics. Bottom row: Synchronized stereo samples of multi-view inconsistency caused by camera occlusions

We post-processed the recorded data into eight sequences containing four targeting (*Dil*) and four endoscopic intervention phases (*End*), with a summary provided in Table 1 (a more detailed breakdown of post-processing steps is provided the Online Resource 1). While *End* sequences were longer, *Dil* phases involved faster motion. Common challenges include the surgeon occluding part of the field of view, surgical staff movement in the background or foreground when assisting the surgeon, or short sections when the camera faces near featureless areas such as the surgical drape, illustrated in the middle row of Fig. 3.

### Performance evaluation

**Table 2:** vSLAM result, reporting the median rms ATE [mm] of three runs. Best performance is highlighted in bold, second best underscored

	Left1		Right1$^*$		Left2		Right2$^*$		
	Dil	End	Dil	End	Dil	End	Dil	End	Mean
*Monocular*									
DSO [20]	1.4	6.6	3.1	50.3	**1**.**4**	15.0	0.9	21.3	12.5
ORB-SLAM3 [19]	2.1	13.6	7.0	11.4	9.3	15.5	2.1	10.3	8.9
DROID-SLAM [21]	1.2	**1**.**6**	1.0	**3**.**4**	4.4	**2**.**2**	0.9	**1**.**1**	**2**.**0**
DPVO [22]	1.0	**1**.**6**	1.2	4.0	33.8	37.6	1.2	2.4	10.4
MASt3R-SLAM [23]	2.5	11.6	7.5	8.9	13.5	6.4	-	6.1	7.1
*Stereo*									
ORB-SLAM3 [19]	20.4	18.4	39.2	28.8	18.6	18.2	13.6	28.7	23.2
DROID-SLAM [21]	**0**.**9**	1.9	**0**.**8**	49.7	**4**.**0**	37.1	**0**.**8**	34.1	18.8

$^*$Optical tracking baseline post-processed due to minor setup issues (See Online Resource 1) The result with the best performance is in bold, and the second best underlined

**Table 3:** vSLAM orientation result, reporting the median angular rms ATE [deg] of three runs. Best performance is highlighted in bold, second best underscored

	Left1		Right1$^*$		Left2		Right2$^*$		
	Dil	End	Dil	End	Dil	End	Dil	End	Mean
*Monocular*									
DSO [20]	1.68	2.68	1.07	52.20	**2**.**07**	6.09	1.48	13.90	10.15
ORB-SLAM3 [19]	1.30	3.29	3.93	**1**.**88**	4.51	2.97	1.61	4.28	3.78
DROID-SLAM [21]	0.99	**0**.**72**	0.51	2.40	3.56	**1**.**11**	0.94	**1**.**49**	**1**.**47**
DPVO [22]	**0**.**88**	**0**.**72**	**0**.**36**	2.06	44.47	111.40	**0**.**39**	1.85	20.27
MASt3R-SLAM [23]	2.36	4.51	3.40	2.70	7.55	3.74	-	2.89	3.88
*Stereo*									
ORB-SLAM3 [19]	6.75	8.65	22.14	12.93	19.23	6.70	18.95	7.63	12.87
DROID-SLAM [21]	1.88	0.75	1.29	9.65	6.65	50.02	1.77	34.40	13.30

$^*$ Optical tracking baseline post-processed due to minor setup issues (See Online Resource 1) The result with the best performance is in bold, and the second best underlined

We present the achieved rms ATE for translation (Table 2) and rotation (Table 3), where a dash indicates that no evaluation could be performed. Overall, DROID-SLAM achieved the best performance across both values, reaching a mean rms ATE of 2.0 mm and 1.47 degrees. It outperformed other methods by a considerable margin, with the next best methods ORB-SLAM3 and MASt3R-SLAM achieving scores of 8.9 mm 3.78 degrees and 7.1 mm 3.88 degrees, respectively. DPVO, generally exhibited similar performance to DROID-SLAM on most sequences but failed to maintain accurate tracking on the Left2 recording due to pronounced motion blur and occlusions. MASt3R-SLAM also showed promising results. However, its evaluation was limited to keyframes, since the available open-source implementation could not provide pose estimates for all frames at the time of writing (leading to insufficient ground truth pose correspondences for trajectory alignment in Right2Dil).

An important finding is that stereo methods generally performed worse than their monocular counterparts on our dataset. ORB-SLAM3 itself reported similar performance differences [19], likely as its monocular mode can take full advantage of the repeated observations, optimizing the map and poses over time, while its stereo mode strongly relies on accurate stereo depth. For stereo ORB-SLAM3, the scale factors estimated during alignment (Eq. 3) had a mean and standard deviation of 0.75±0.16, indicating insufficient scale accuracy. DROID-SLAM showed a greater ability to leverage the imperfect stereo observations without introducing scale drift, but still failed to correctly estimate the scale (scale factor of 0.68±0.37). The algorithm especially struggled in handling the longer *End* sequences, where camera occlusions caused severe multi-view inconsistencies (see Fig. 3, bottom row).

Furthermore, we observed a general difference between the tracking accuracy of the short *Dil* sequences compared to the long *End* ones, with the top-performing methods being comparatively less affected by the sequence length. The long sequences increased the risk of tracking failure, in which case relocalization would be necessary. Among all methods, learning based approaches were the most stable, all tracking continuously. On average, ORB-SLAM3 tracked 96% (98% in stereo mode), while DSO only successfully tracked 74% of the frames. A full table, trajectory examples, and additional results are provided in Online Resource 1.

### Discussion

Among the evaluated methods, DROID-SLAM achieved the lowest translational and rotational ATE on average, outperforming the performance reported in prior work [8], even in realistic surgical conditions. While no formal accuracy threshold for clinical acceptability for navigation-assisted spine surgery exists [26], commonly referenced standards such as the Gertzbein-Robbins grading scheme for pedicle screw placement [27] consider breaches up to 2 mm (Grade B) to be acceptable. Under this reference, DROID-SLAM achieved acceptable accuracy in the majority of sequences, suggesting potential feasibility.

While DROID-SLAM consistently outperformed other methods and demonstrated superior reliability in handling even cases with pronounced occlusions, motion blur, and scene-object dynamics, its performance still degrades under more challenging conditions. Notably, while background dynamics could be robustly handled, trajectories containing dynamic motion in the foreground or strong occlusions showed a performance decline. Similar limitations were observed in the stereo setup, where partial occlusions in one of the camera views posed a significant challenge. Although stereo could theoretically be used to solve the scale ambiguity of the monocular approach, the stereo depth of our off-the-shelf setup was insufficient. These findings highlight the need for an improved sensor setup and further stereo-specific vSLAM improvements to enable reliable intraoperative deployment.

Our collected, publicly available dataset provides a standardized benchmark for such future methods, allowing a direct evaluation under realistic surgical conditions. Therefore, we directly address the lack of a clinically relevant dataset for inside-out tracking, bridging the gap between general computer vision and practical intraoperative implementations.

Nonetheless, several limitations must be acknowledged. The sample size was limited within this dataset, restricting the generalizability of the findings. Furthermore, the experiments were conducted on a high-fidelity training phantom under controlled laboratory conditions, which cannot fully replicate in vivo anatomical and environmental variability. In real endoscopic spine surgery, we would generally expect a more cluttered and dynamic environment, e.g., caused by additional surgical staff assisting during an intervention, or by a patient’s breathing motion, which can decrease the performance.

Despite mentioned limitations, our collected clinical-grade dataset highlights potential for advancing inside-out tracking toward routine use in navigated spinal endoscopy. Future research shall focus on improving reliability and generalizability through the following potential directions:

- Collecting additional data of different endoscopic spine surgeries to increase variability and assert generalizability.
- Testing and improving custom stereo and multi-camera SLAM systems to handle multi-view inconsistencies and ensure accurate scale estimation.
- Exploring visual inertial SLAM to handle motion blur and occlusions robustly.
- Developing dynamic SLAM methods for the surgical environment, to identify and prioritize static regions.

## Conclusion

In this study we aimed at laying out the groundwork for inside-out surgical instrument tracking using tool-mounted cameras, with a particular focus on endoscopic spine surgery. Our early benchmarking results demonstrated that state-of-the-art algorithms such as DROID-SLAM [21] can achieve an overall average rms ATE of 2 mm and 1.47 degrees, indicating strong promise for accurate intraoperative tool tracking. Beyond these benchmarking results, our collected dataset was proven valuable in revealing key environmental and operational factors that still limit robustness under realistic surgical conditions. These insights highlight the importance of continued research toward improving reliability, and clinical integration of inside-out tracking for navigated spinal endoscopy.

## Supplementary Information

Below is the link to the electronic supplementary material.

Supplementary file 1 (pdf 7009 KB)

## Footnotes

1 https://github.com/luxonis/depthai
