# Supplementary material for: Markerless inside-out tool tracking for endoscopic spine surgery: a benchmarking study and clinical dataset
Source: Int J Comput Assist Radiol Surg. 2026 Apr 27;21(5):1053–60. doi: 10.1007/s11548-026-03625-z (PMC13687307; doi:10.1007/s11548-026-03625-z)
Supplement: Supplementary file 1 [file 11548_2026_3625_MOESM1_ESM.pdf]

Online Resource 1

Markerless Inside-Out Tool Tracking for Endoscopic Spine Surgery: A Benchmarking Study and Clinical Dataset

Peter Zhang, Youyang Shen, Frédéric Giraud,
Nicola A. Cavalcanti, Marc Pollefeys, Christoph J. Laux,
Philipp Fürnstahl, Hooman Esfandiari

Journal:
International Journal of Computer Assisted Radiology and Surgery (IJCARS)

Affiliations of the corresponding author:
Computer Vision and Geometry Group, ETH Zurich, Zurich, Switzerland
Research in Orthopedic Computer Science, University of Zurich, Zurich, Switzerland

Corresponding Author: Peter Zhang
E-mail: pzhang@ethz.ch

1 Dataset

In this section, additional information on the ground truth and general information on the endoscope recordings are provided.

1.1 Ground Truth Accuracy

The provided ground truth consists of the chained transformations provided by the fusionTrack™500 (Atracsys, Switzerland) optical tracking system and our hand-eye calibration. In this section, we quantify the accuracy of each.

Optical Tracking Accuracy: For our physical setup (tracking camera placed 1.8 m to 2.4 m away from the marker), the manufacturer reports a root mean squared (rms) fiducial localization error (FLE) of 0.11 mm[1]. As the resulting target registration error (TRE) is highly non-linear and depends on the exact marker geometry, we do not report the theoretical expected error following [1, 2], but performed a Monte Carlo simulation using our specific marker geometry. The 3D marker detection errors were modeled as independent and identically distributed (IID) Gaussian noise with zero mean and standard deviation of 0.11 mm. The resulting rigid registration of the known marker geometry showed a translation offset of 0.31 ± 0.17 mm and an orientation offset of 0.29 ± 0.13 degrees.

Hand-eye Calibration Accuracy: To obtain a metric measure of the hand–eye calibration accuracy between the tracked marker and the camera frame, we conducted a Monte Carlo simulation. Checkerboard corner detection errors were modeled as IID Gaussian noise with zero mean and a standard deviation of 1.6 px, corresponding to the average reprojection error observed during calibration. Using the same setup as in the actual hand–eye calibration (namely, the pinhole camera model, checkerboard geometry, and number of poses), we simulated calibration runs by randomly sampling camera poses at a distance of 50 cm from the checkerboard within a 30 degree cone. The resulting calibration accuracy showed a translation error of 0.27 ± 0.14 mm and a rotation error of 0.033 ± 0.012 degrees.

1.2 Ground Truth Processing

In the dataset, we provide the filtered ground truth. The raw tracking data is cut according to the sequence length, and timesteps for which the registration error exceeds 0.5 mm are filtered out. Additionally, it is important to note that there were minor mistakes during the data capture of the endoscopic spine surgeries from the right side, which had to be post-processed to enable a meaningful evaluation. In the first recording from the right side (RightDil1 and RightEnd1), the Precision Time Protocol (PTP) synchronization of the optical tracking system was not set up correctly, causing a time offset. During the second recording (RightDil2, RightEnd2), the C-arm was initially blocking the line-of-sight to the tracked tools, prompting us

[1] https://atracsys.com/product/fusiontrack-500/

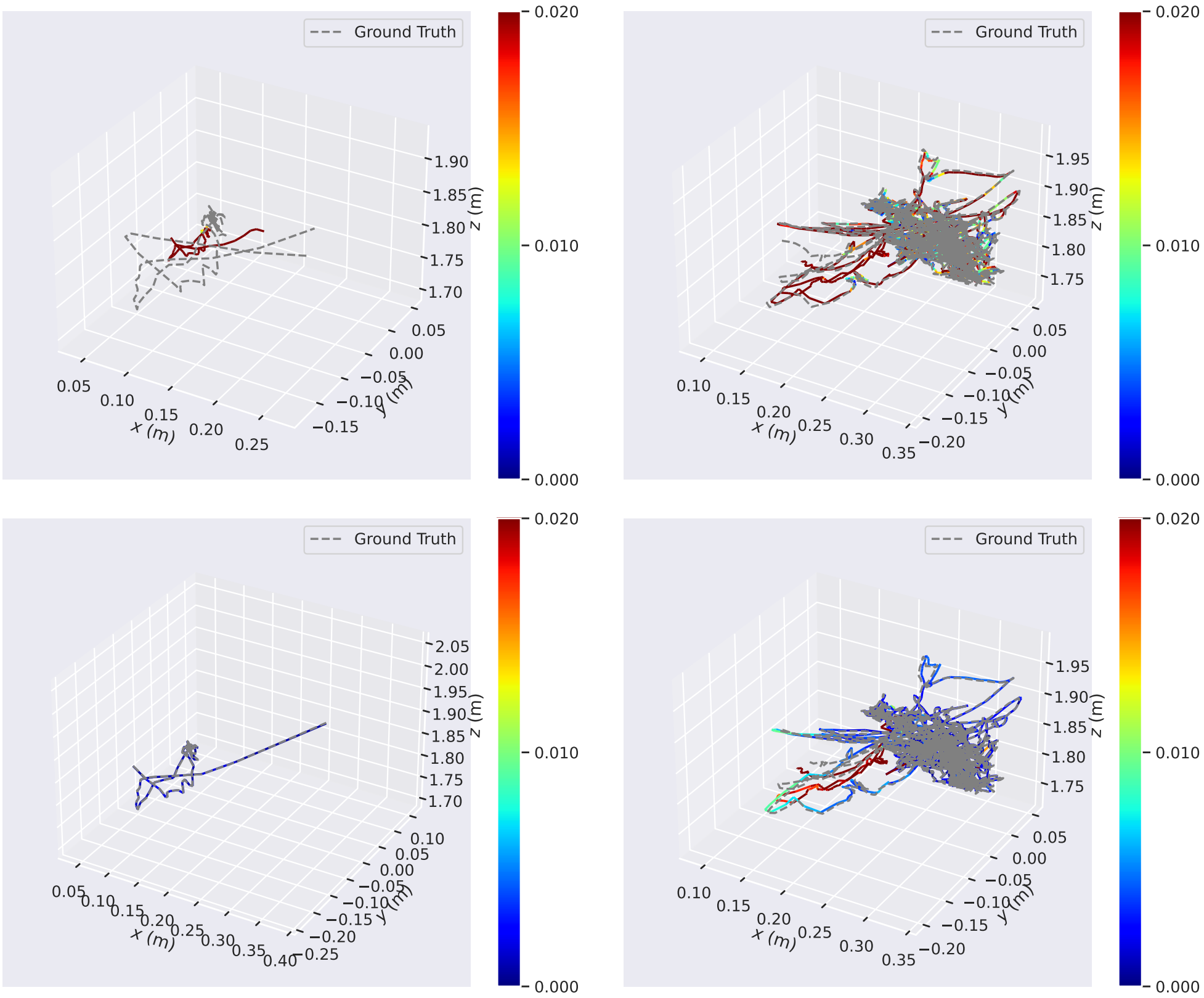

Fig. 1: The two trajectories on the left show the RightDil1 and RightEnd1 trajectories (ground truth in gray, estimated trajectory by DROID-SLAM [4] colored according to the rms ATE in m) before adjusting the time offset for alignment, while the right side shows the same trajectories after alignment.

to move the optical tracking system during the recording.

Offset estimation: To estimate the constant offset that occurred within the Right1 recording, we defined a cost function as the mean root mean squared absolute trajectory errors (rms ATE) of all estimated trajectories by the compared methods, given an alignment using Umeyama's method [3] with the time offset as the scalar optimization parameter. Non-linearly solving for the minimum using the Nelder-Mead method, produced an estimated 0.548 s offset. We are aware that the results following this temporal alignment are biased and need to be taken with a grain of salt. We show the obvious misalignment in the first row of Figure 1, and after the adjustment on the second row. Note that the same optimization applied to another well-tracked recording (e.g., Left1) results in a sub-millisecond adjustment, barely affecting the evaluation.

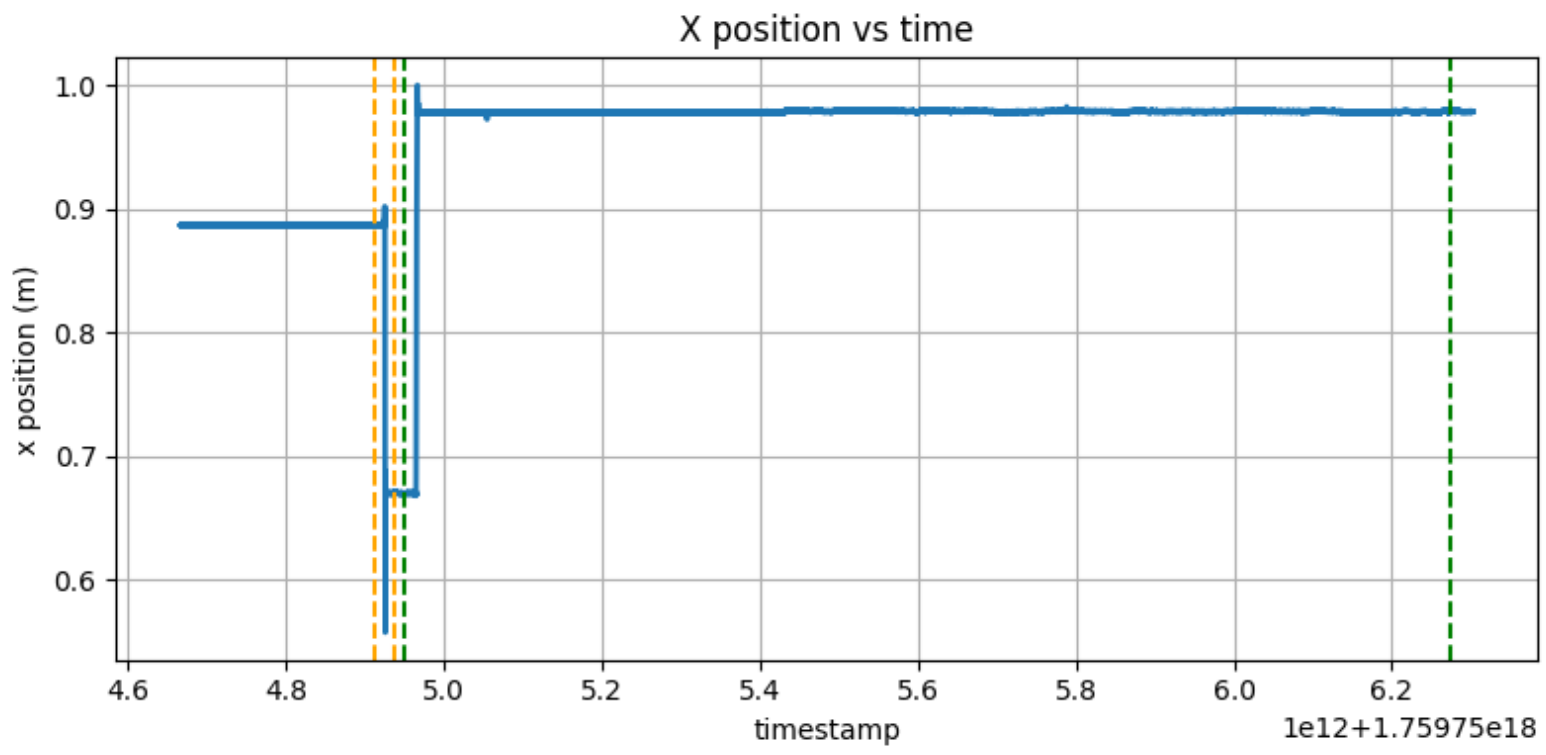

Fig. 2: The tracked x-axis coordinate of the surgical training model in blue. As it was static, this trajectory can be interpreted as the motion of the optical tracking system. Orange indicates the *Dil* sequence and green the *End* sequence.

Motion Removal: Within the Right2 recording, the optical tracking system had to be moved twice due to objects blocking the line-of-sight of the optical tracking system, causing the raw tracking data to produce erroneous ground truth. As the surgical model remained static during the whole recording, we manually segment the clearly visible sections, analyzing its trajectory shown in Figure 2. The evaluation was thus performed on a reduced set of poses for these sequences. Specifically for the RightDil2 sequence, only around 44% of the trajectory contains valid ground truth, while around 98% is available for the RightEnd2 sequence.

1.3 Endoscopic Feed

The endoscopic feed was not utilized for the inside-out tracking evaluation, where only the tool-mounted camera feed was used by the compared methods. Nonetheless, we recorded, calibrated, and synchronized the endoscopic feed for future developments. We recorded the endoscopic feed by capturing the HDMI output at a resolution of 1920x1080 and a framerate of 60Hz. Example frames are shown in Figure 3. The timestamp was assigned once a custom ROS2 node received the each frame. To synchronize the endoscopic feed to the host clock, we recorded a common time reference RocSync [5] with the Luxonis Oak-D Pro W camera and the endoscope itself. The observed reference acts as an independent clock, showing its time from start with millisecond accuracy using LEDs. Using the observed timestamp difference between each image stream and the reference clock, we estimate a constant offset. Note that the frame timestamp was assigned by a periodically spinning ROS2 node, and that we observed fast-changing exposure times of the endoscope, limiting the reliability of our time offset estimation approach. As we used a 30° angled endoscope, the calibration of the endoscopic view was not trivial. We present the calibration approach in the following section.

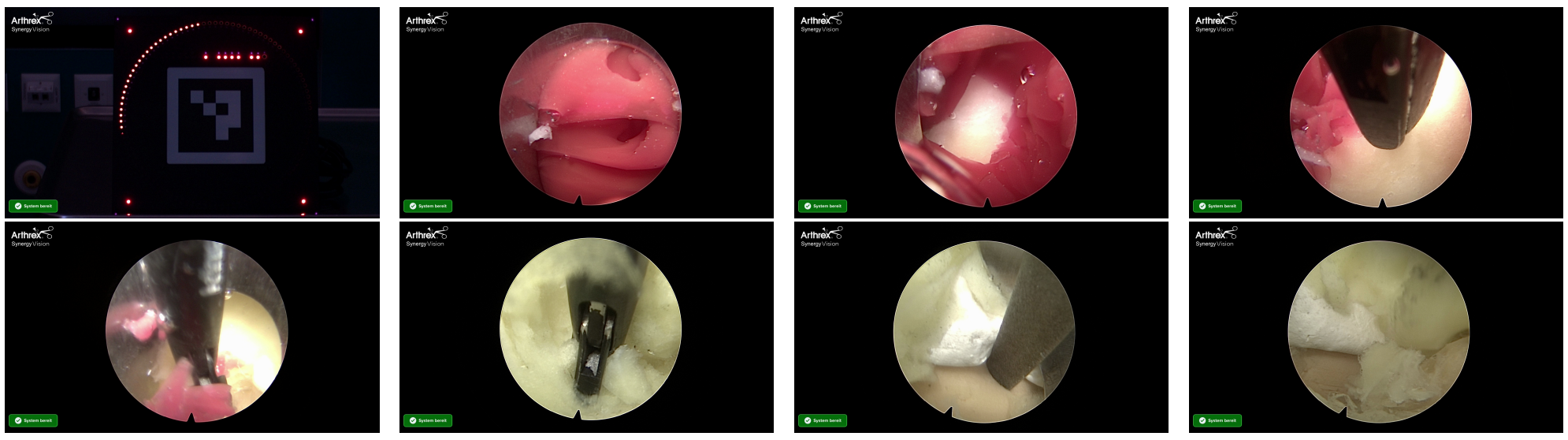

Fig. 3: Example frames from the endoscope. The top left shows the external time reference RocSync used for synchronization, while the rest, from left to right and top to bottom, depict the scene during the endoscopic intervention.

2 Endoscope Calibration

For the calibration of the angled endoscope, we mainly followed the models proposed in [6] and [7]. We mounted passive infrared markers directly onto the endoscope optics, tracking its rigid motion while allowing free rotation of the camera head. Using the wedge-shaped notch within the endoscopic images, we extract the current rotation angle θ of the camera head. The model proposed in [6] to get the projected points x_i given its 3D coordinates X_i is as follows:

$$\lambda x_i' = K \cdot {}^{C_e}T_{M_{End}} \cdot {}^{M_{End}}T_{OT} \cdot {}^{OT}X_i \tag{1}$$

$$x_i = R(\theta)(x_i' - cc) + cc, \tag{2}$$

where ${}^{M_{End}}T_{OT}$ is the transformation measured by the optical tracking system, ${}^{C_e}T_{M_{End}}$ a hand-eye calibration of the marker to the endoscope camera, K the intrinsic camera matrix, and cc the principal point of the intrinsics. Decoupling the endoscope calibration into the camera intrinsics, the rigid hand-eye calibration, and a rotation θ of the camera head. In this work, we confirm the observations from [7, 8], namely that the principal point is not static, but dependent on θ. We thus adapted equation 2, and use the following:

$$x_i = R(\theta)(x_i' - cc(\theta)) + cc(\theta), \tag{3}$$

with the exact expression for $cc(\theta)$ explained in the following sections. A general schematic overview of the coordinate frames and transformations involved is shown in Figure 6a.

2.1 Camera Intrinsics

We use the camera calibration method by Zhang [9] on endoscopic frames, where a small checkerboard with an edge length of 5 mm is visible, modeling the endoscopic camera as a pinhole camera with radial distortion (considering only parameters k_1, k_2). Repeating the calibration for different camera angles θ, we observed that the focal lengths f_x, f_y and distortion coefficients k_1, k_2 are approximately constant, while the

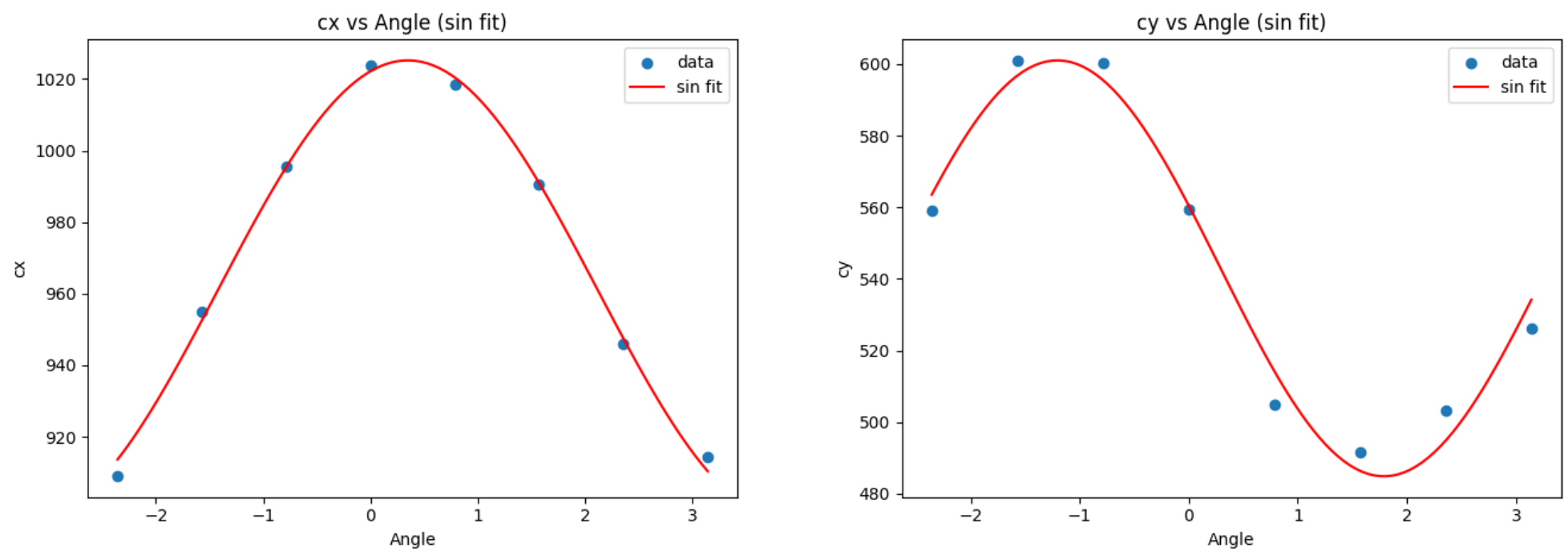

(a) (b)

Fig. 4: The sinusoidal fit of the principal point, dependent on the angle θ.

principle point cc_x, cc_y follows a sinusoidal pattern. Using the intrinsic calibration values from eight different angles, we fit the sinusoidal function:

$$cc_{x,y}(\theta) = a_{x,y} sin(b_{x,y}\theta + \phi_{x,y}) + d_{x,y}, \tag{4}$$

where a, b, ϕ, d are the fitted constants, which we provide in the dataset. Figure 4 shows the fitted sinusoids, dependent on the angle θ.

2.2 Angle Detection

The angle θ is detected by processing each endoscopic frame. First the boundary contour is estimated by sampling from a thresholded frame, fitting a circle using RANSAC. In a second step, the candidate contours are extracted within the detected circle boundary, computing geometric features (area, distance to circle center, area to perimeter ratio) for each. If defined thresholds are met, the candidate is considered as the notch. Using the notch centroid and the detected circle center, the angle can be calculated. This pipeline is visualized in Figure 5, while we also an illustration of the angle-dependent principle point based on the detected notch and circle center in Figure 6b.

2.3 Hand-eye Calibration

For hand-eye calibration ${}^{C_e}T_{M_{End}}$ between the endoscope camera and the attached markers, we use Tsai's method [10], which leverages ${}^{OT}T_{M_{End,i}}$ and ${}^{Check}T_{C_e,i}$ to estimate the rigid transformations between C_e and M_{End}, as well as between OT and $Check$. The endoscope camera to checkerboard transformations ${}^{Check}T_{C_e,i}$ for each image i is estimated using PnP, while the optical tracking system OT observes the marker M_{End} pose. We utilize the output from Tsai's methods as an initial guess and

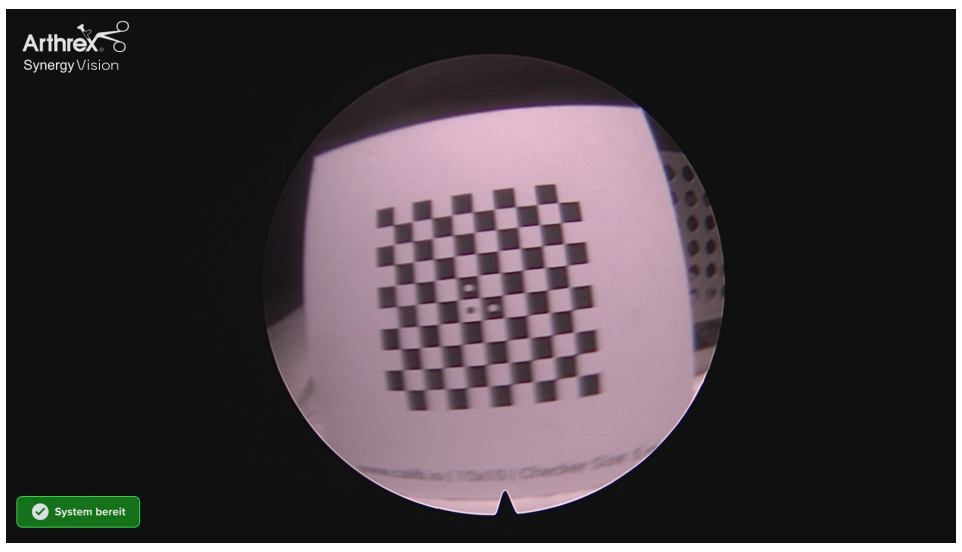

(a) Raw endoscopic image.

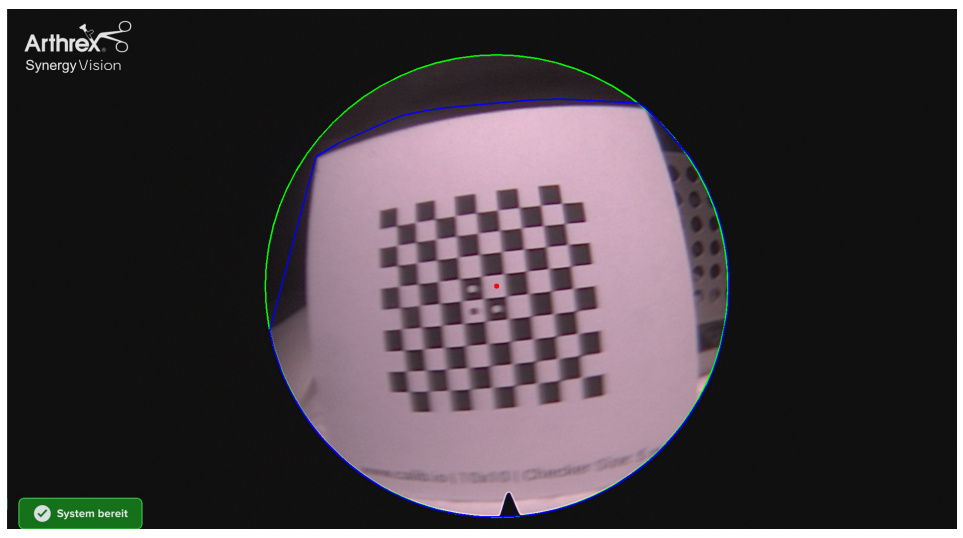

(b) Boundary contour extraction and RANSAC-based circle fitting. Blue contours represent thresholded boundaries; the green circle indicates the estimated scope boundary.

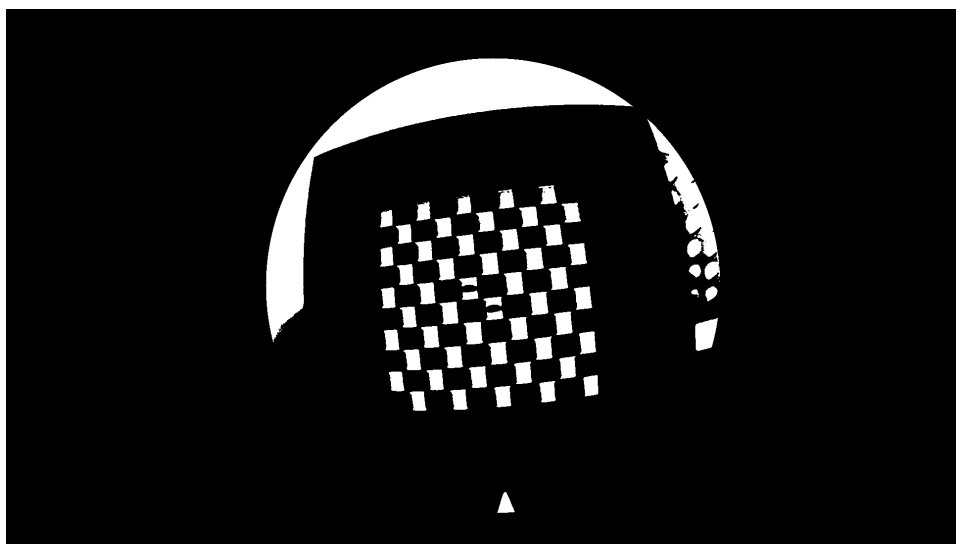

(c) Circular mask applied to isolate the valid imaging region.

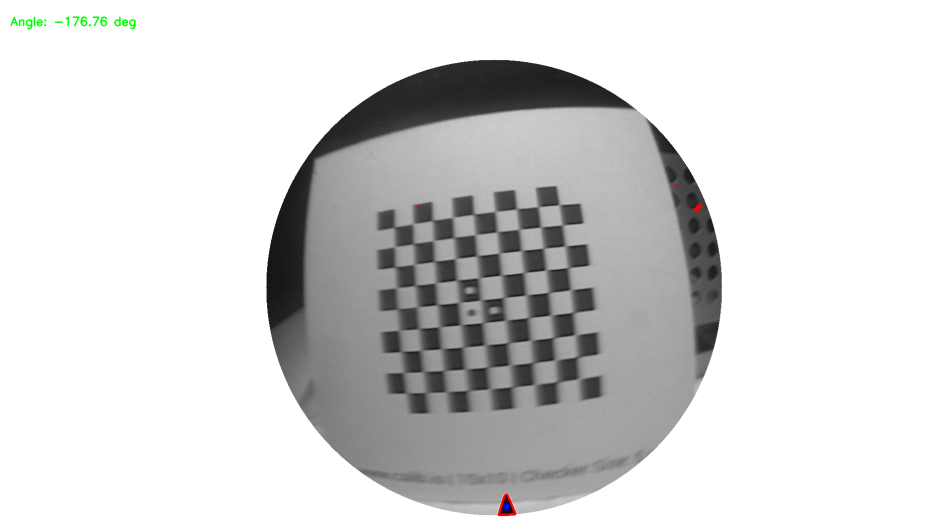

(d) Detected notch based on geometric thresholding using area, centroid distance, and area-to-perimeter ratio.

Fig. 5: Visualization of the proposed rotation estimation process. (a) Raw image. (b) Boundary contour extraction and circle fitting. (c) Masked endoscopic region. (d) Notch detection using geometric constraints.

non-linearly refine it by optimizing the following cost:

$$^{C_e}\hat{T}_{M_{End}} = argmin_{\xi} \sum_{i=1}^{N} \sum_{j=1}^{C} \left| x_j - \Pi(X_j, \xi) \right|_2^2. \tag{5}$$

For all checkerboard corners $j \in [1, C]$ and samples $i \in [1, N]$, the reprojection error is calculated, with $\Pi(X_j, \xi)$ projecting the point X_j onto the image plane. ξ is the vector of optimization parameters that contains rigid transformations $^{C_e}T_{M_{End}}$ and $^{OT}T_{Check}$.

2.4 Evaluation

The intrinsic camera calibration, using constant values for the focal length and distortion coefficients and fitted principal point, achieves reprojection errors below 0.1 pixels

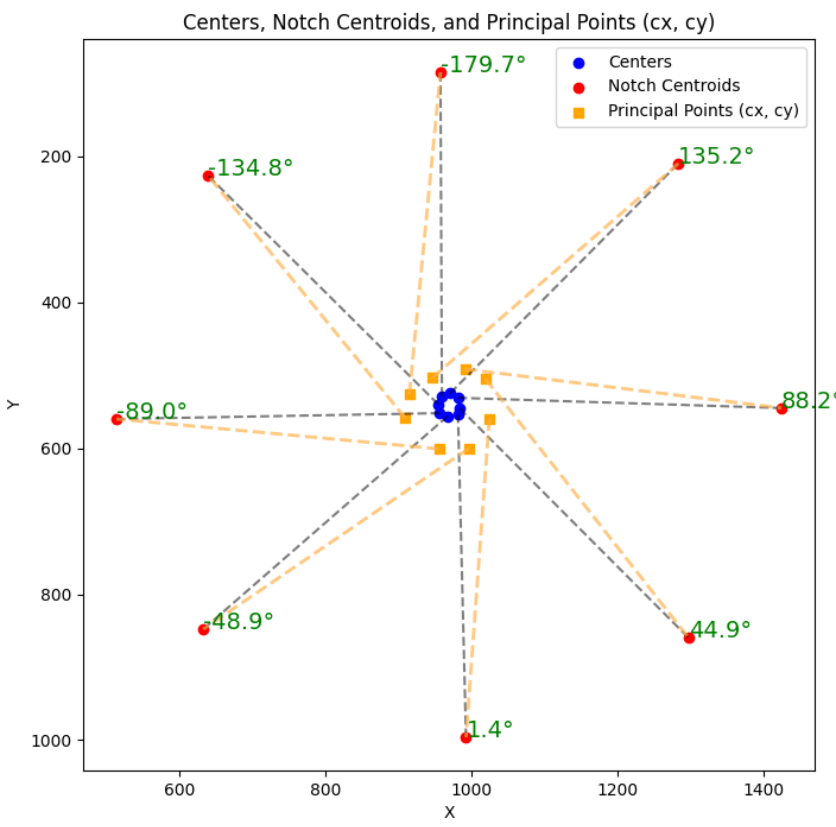

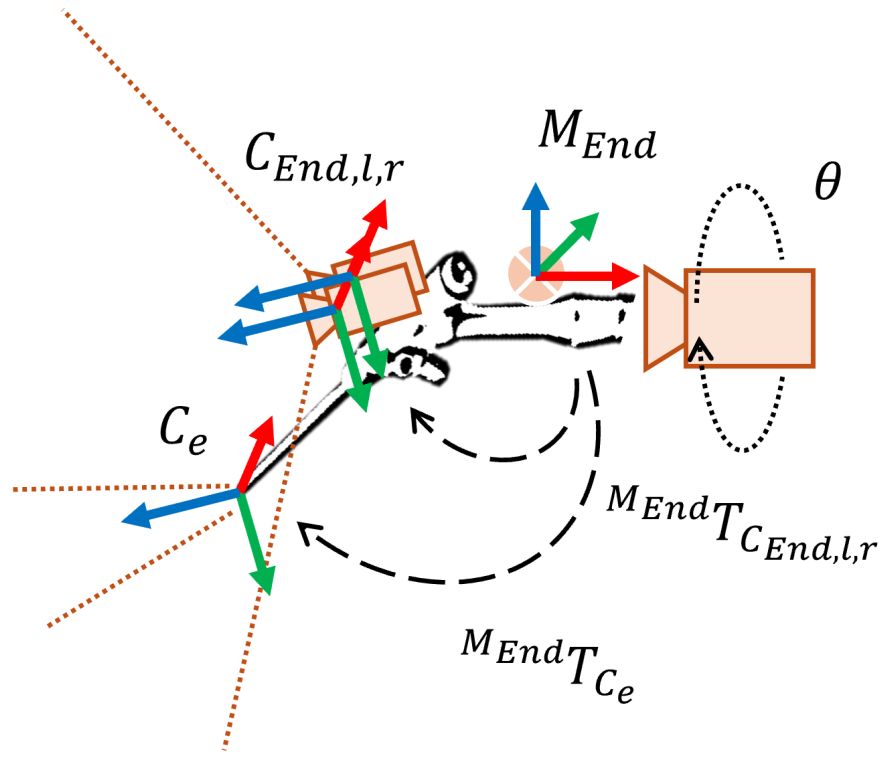

(a) (b)

Fig. 6: **(a)** Shows the principal points (orange), the detected notch (red), and the circle centers (blue) for 8 different view angles. **(b)** Illustrates the endoscope setup with the calibrated rigid transformations and the angle θ.

for all eight evaluated angles. Using an optically tracked checkerboard, we evaluate the whole geometric transformation chain of the endoscope at various θ angles, following equations 1 and 3. The achieved mean reprojection error across all angles is 10.07 ± 3.33 pixels. We show the reprojection error depending on the angle in Figure 7, and qualitative results from this evaluation in Figure 8. The reprojection error is relatively large, depending on the endoscope angle. We observed a non-negligible impact from the insufficient synchronization accuracy (obvious angle offset during fast rotations), as well as a high sensitivity to the hand-eye calibration, given the small scale of the endoscope images compared to the marker setup.

3 Results

In this section, we provide additional details on the evaluations performed.

3.1 Non-deterministic Behaviour

To account for the non-deterministic behaviour of the visual simultaneous localization and mapping (vSLAM) methods, each method was run three times, and the median was reported in the main paper. We provide a full color-coded evaluation of each execution in Figure 9. The learning-based methods DROID-SLAM, DPVO [11], and MASt3R-SLAM [12] were stable across different executions, with the exception of stereo DROID-SLAM showing larger variations in a few sequences. DSO [13] did not

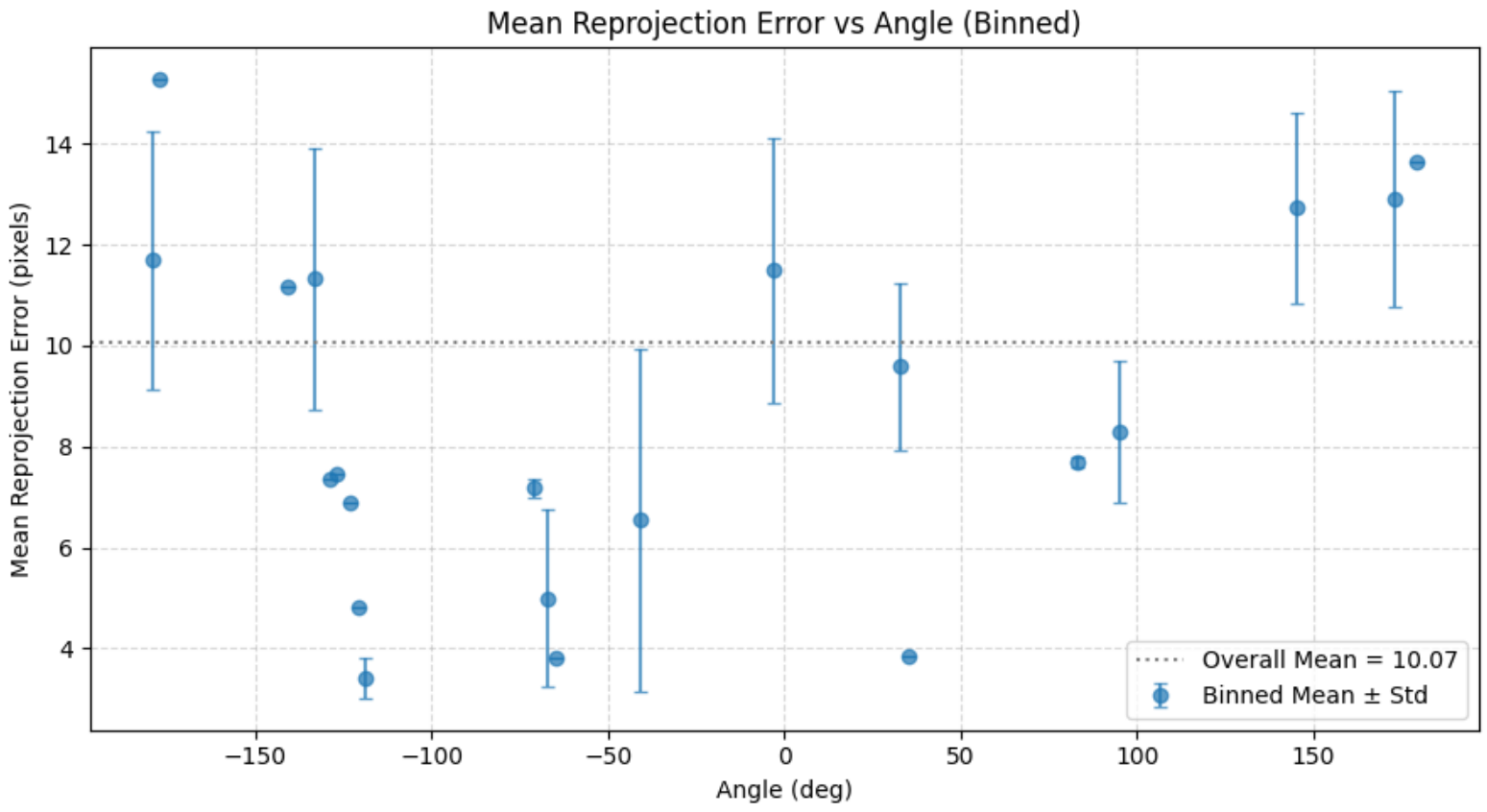

Fig. 7: Reprojection error depending on the angle θ.

show large performance differences depending on the run either, similar to ORB-SLAM3 [14] in monocular mode, whereas the stereo mode of ORB-SLAM3 showed major fluctuations between executions.

3.2 Tracking Rate

Defining the tracking rate as the time difference between the first frame and the last frame, divided by the total trajectory length, we present the full table of tracking rates of all compared methods on each sequence in Table 1. Learning-based methods always estimate a pose, leading to 100% tracking rate across the board. The exception is MASt3R-SLAM, as it only saves the sparse keyframe poses. DSO and ORB-SLAM3 can loose track during challenging sequences without being able to relocalize.

Note that we can provide a tracking rate quantification for the Right2Dil sequence, as MASt3R-SLAM was able to track it. The rms ATE was not reported in the main paper, since the method provided only three keyframe poses, where one did not correspond to a ground truth pose due to the data collection issue mentioned above.

Table 1: vSLAM Result, fraction of the total trajectory tracked.

	Left1		Right1		Left2		Right2		
	Dil	End	Dil	End	Dil	End	Dil	End	Mean
Monocular									
DSO [13]	0.89	1.00	0.96	0.71	0.91	0.24	0.98	0.25	0.74
ORB-SLAM3 [14]	0.90	0.99	0.92	1.00	0.84	1.00	0.99	1.00	0.96
DROID-SLAM [4]	1.00	1.00	1.00	1.00	1.00	1.00	1.00	1.00	1.00
DPVO [11]	1.00	1.00	1.00	1.00	1.00	1.00	1.00	1.00	1.00
MASt3R-SLAM [12]	0.99	1.00	0.98	0.98	0.99	1.00	0.97	0.99	0.99
Stereo									
ORB-SLAM3 [14]	0.98	0.99	0.97	1.00	0.93	1.00	1.00	0.99	0.98
DROID-SLAM [4]	1.00	1.00	1.00	1.00	1.00	1.00	1.00	1.00	1.00

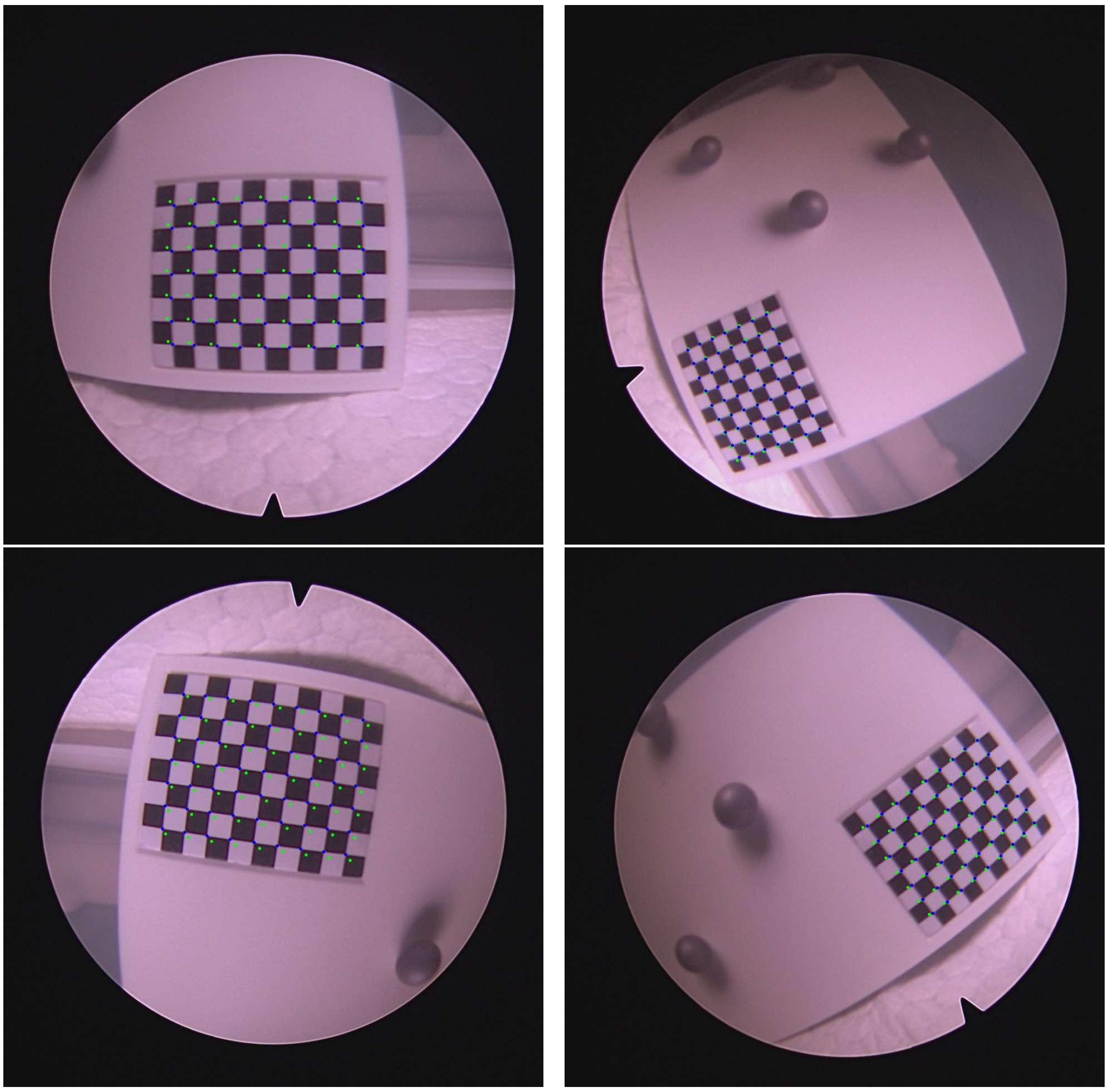

Fig. 8: Qualitative example images from the projection of the checkerboard corners onto the image plane. The blue points are the detected corners, while the green points are the checkerboard coordinates projected using the entire transformation chain.

3.3 Stereo Scale

The evaluated stereo vSLAM methods generally underperformed compared to their monocular counterparts when considering the rms ATE after alignment using Umeyama's method [3]. The corresponding scale factors, which reflect the scale estimation error of each method, are reported in Table 2. Neither ORB-SLAM3 nor DROID-SLAM in stereo mode was able to reliably recover the correct scale, likely due to the small stereo baseline and the frequent re-observation of the same scene.

As shown in Table 3, DROID-SLAM can still achieve comparatively low rms ATE values after scale alignment for some sequences, in contrast to ORB-SLAM3. This suggests that DROID-SLAM is less sensitive to inaccurate stereo depth estimates and

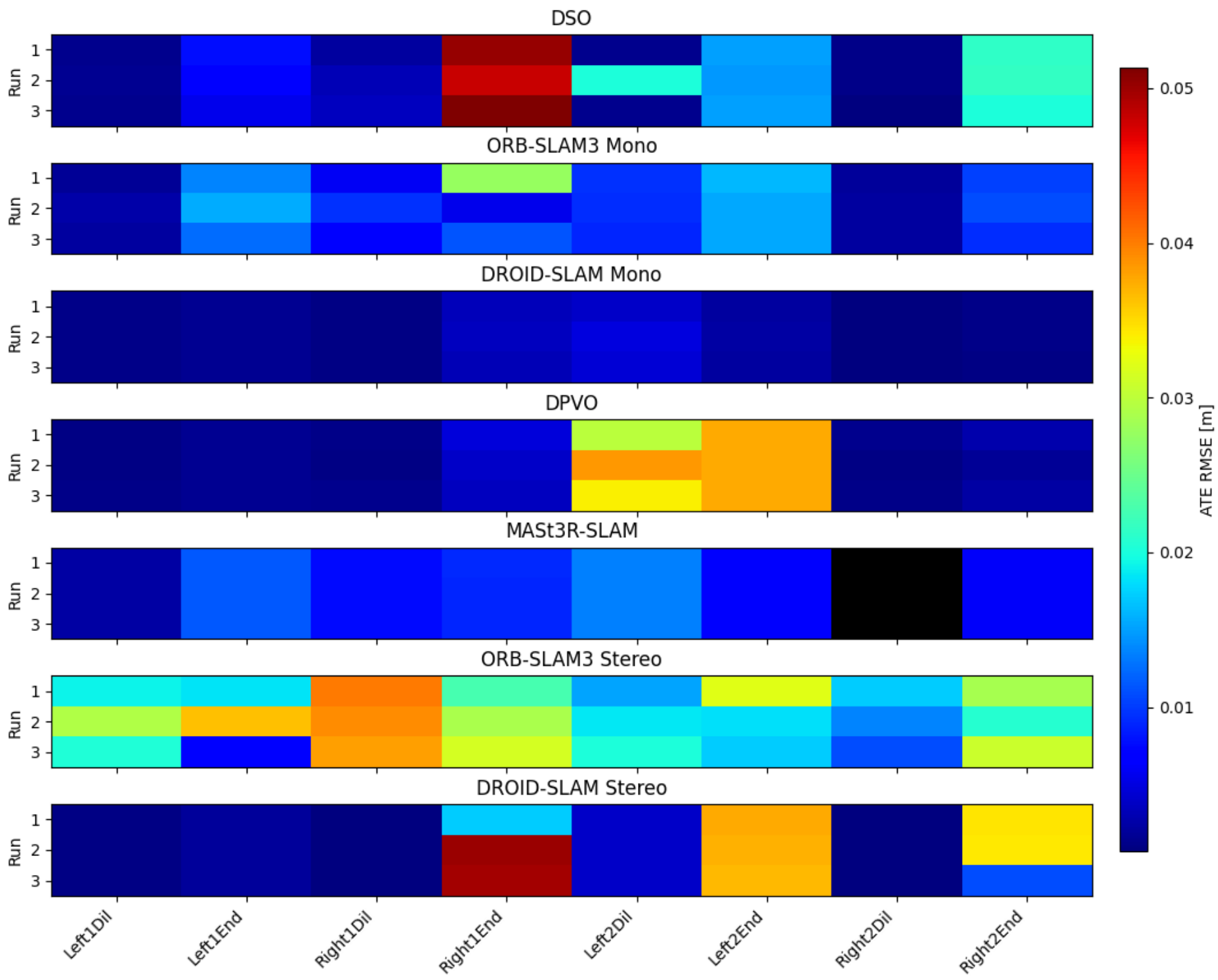

Fig. 9: Color-coded plot showing the rms ATE of each execution.

Table 2: Alignment scale factor used for stereo vSLAM alignment in the main paper.

	Left1		Right1		Left2		Right2		
	Dil	End	Dil	End	Dil	End	Dil	End	Mean
Stereo									
ORB-SLAM3 [14]	0.73	0.92	0.74	0.78	0.44	0.88	0.92	0.61	0.75
DROID-SLAM [4]	0.91	0.92	0.93	0.04	0.91	0.63	0.91	0.18	0.68

is more capable of maintaining scale consistency. However, the rms ATE evaluated without scale alignment (Table 3) still indicates that neither method estimates the absolute scale accurately enough for a real use-case.

3.4 Qualitative Results

To give an overview of the trajectories and the tracking accuracy achieved by the best performing method DROID-SLAM, we plot the ground truth and estimated trajectory of all sequences in Figure 10. Overall, the estimated trajectories show good alignment

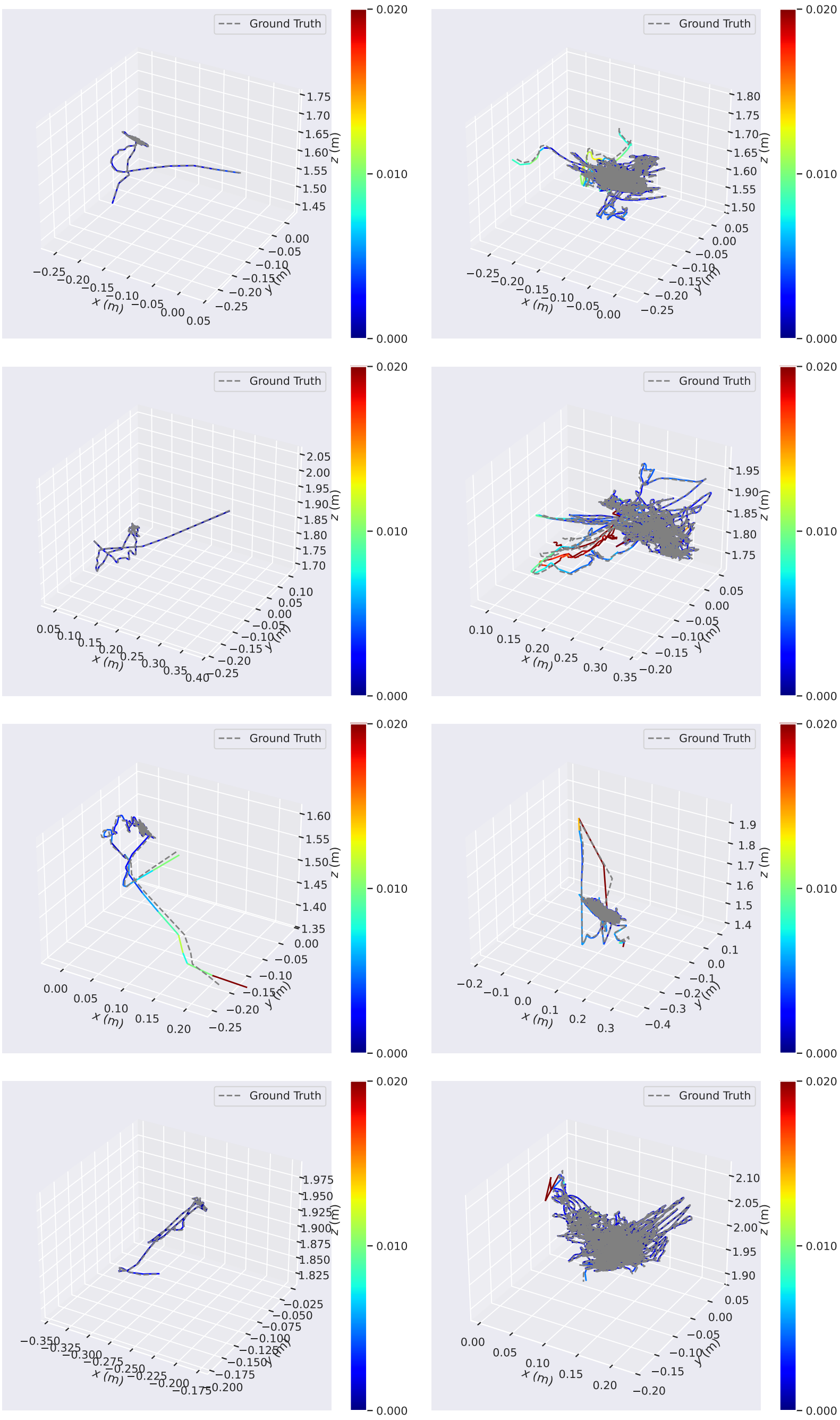

Fig. 10: The estimated trajectories of DROID-SLAM [4] (monocular) compared to the ground truth, with color-coded error. The sequences shown correspond to the order within Table 1, starting from the top left, ending at the bottom right.

Table 3: Stereo vSLAM Result, median rms ATE with and without scale alignment.

	Left1		Right1		Left2		Right2		
	Dil	End	Dil	End	Dil	End	Dil	End	Mean
w/ scale alignment									
ORB-SLAM3 [14]	20.4	18.4	39.2	28.8	18.6	18.2	13.6	28.7	23.2
DROID-SLAM [4]	0.9	1.9	0.8	49.7	4.0	37.1	0.8	34.1	18.8
w/o scale alignment									
ORB-SLAM3 [14]	24.5	18.7	21.4	20.6	66.3	29.4	19.8	35.2	29.5
DROID-SLAM [4]	4.1	4.2	5.5	266.5	5.8	51.4	4.6	159.4	62.7

with the ground truth. Deviations are typically local rather than global, with accuracy degradation occurring in short trajectory segments. These segments are especially pronounced in all less accurately tracked sequences. However, also overall well-tracked sequences, such as Right2End (bottom right, rms ATE of 1.1 mm), can exhibit brief tracking inaccuracies. Minor deviations can be attributed to uncompensated drift, while larger errors are likely caused by visually challenging sections.

References

[1] Fitzpatrick, J.M., West, J.B., Maurer, C.R.: Predicting error in rigid-body point-based registration. IEEE Transactions on Medical Imaging **17**(5), 694–702 (1998) https://doi.org/10.1109/42.736021 . Accessed 2026-01-13

[2] West, J.B., Maurer, C.R.: Designing optically tracked instruments for image-guided surgery. IEEE Transactions on Medical Imaging **23**(5), 533–545 (2004) https://doi.org/10.1109/TMI.2004.825614 . Accessed 2026-01-13

[3] Umeyama, S.: Least-squares estimation of transformation parameters between two point patterns. IEEE Transactions on Pattern Analysis and Machine Intelligence **13**(4), 376–380 (1991) https://doi.org/10.1109/34.88573

[4] Teed, Z., Deng, J.: Droid-slam: Deep visual slam for monocular, stereo, and rgb-d cameras. In: Advances in Neural Information Processing Systems, vol. 34, pp. 16558–16569 (2021). https://proceedings.neurips.cc/paper_files/paper/2021/file/89fcd07f20b6785b92134bd6c1d0fa42-Paper.pdf

[5] Meyer, J., Giraud, F., Wüthrich, J., Pollefeys, M., Fürnstahl, P., Calvet, L.: Roc-Sync: Millisecond-Accurate Temporal Synchronization for Heterogeneous Camera Systems. arXiv. arXiv:2511.14948 [cs] (2025). https://doi.org/10.48550/arXiv.2511.14948

[6] Wu, C., Jaramaz, B., Narasimhan, S.G.: A full geometric and photometric calibration method for oblique-viewing endoscopes. Computer Aided Surgery: Official Journal of the International Society for Computer Aided Surgery **15**(1-3), 19–31

(2010) https://doi.org/10.3109/10929081003718758

[7] Holak, K., Kohut, P., Stangel-Wojcikiewicz, K., Kabzińska-Turek, M., Florczak, J., Petko, M.: Oblique-viewing endoscope calibration in the diagnostics and treatment in the pelvis minor area. Bio-Algorithms and Med-Systems **12**(3), 133–140 (2016) https://doi.org/10.1515/bams-2016-0013

[8] Melo, R., Barreto, J.P., Falcao, G.: A new solution for camera calibration and real-time image distortion correction in medical endoscopy–initial technical evaluation. IEEE Transactions on Biomedical Engineering **59**(3), 634–644 (2012) https://doi.org/10.1109/TBME.2011.2177268

[9] Zhang, Z.: A flexible new technique for camera calibration. IEEE Transactions on Pattern Analysis and Machine Intelligence **22**(11), 1330–1334 (2000) https://doi.org/10.1109/34.888718

[10] Tsai, R.Y., Lenz, R.K.: A new technique for fully autonomous and efficient 3d robotics hand/eye calibration. IEEE Transactions on Robotics and Automation **5**(3), 345–358 (1989) https://doi.org/10.1109/70.34770

[11] Teed, Z., Lipson, L., Deng, J.: Deep patch visual odometry. In: Advances in Neural Information Processing Systems, vol. 36, pp. 39033–39051 (2023). https://proceedings.neurips.cc/paper_files/paper/2023/file/7ac484b0f1a1719ad5be9aa8c8455fbb-Paper-Conference.pdf

[12] Murai, R., Dexheimer, E., Davison, A.J.: Mast3r-slam: Real-time dense slam with 3d reconstruction priors. In: 2025 IEEE/CVF Conference on Computer Vision and Pattern Recognition (CVPR), pp. 16695–16705 (2025). https://doi.org/10.1109/CVPR52734.2025.01556

[13] Engel, J., Koltun, V., Cremers, D.: Direct Sparse Odometry. IEEE Transactions on Pattern Analysis and Machine Intelligence **40**(3), 611–625 (2018) https://doi.org/10.1109/TPAMI.2017.2658577

[14] Campos, C., Elvira, R., Rodríguez, J.J.G., M. Montiel, J.M., D. Tardós, J.: ORB-SLAM3: An Accurate Open-Source Library for Visual, Visual–Inertial, and Multimap SLAM. IEEE Transactions on Robotics **37**(6), 1874–1890 (2021) https://doi.org/10.1109/TRO.2021.3075644
